# Supplementary material for: Multi‐dataset identification of innovative feature genes and molecular mechanisms in keratoconus
Source: J Cell Mol Med. 2024 Sep 19;28(18):e70079. doi: 10.1111/jcmm.70079 (PMC11412914; doi:10.1111/jcmm.70079)
Supplement: Supplementary file 2 — Material S2. [file JCMM-28-e70079-s002.zip › 7.GSEA analysis/3.DBNDD1/KEGG_ADHERENS_JUNCTION.html]

Details for gene set KEGG\_ADHERENS\_JUNCTION[GSEA]

|  || Dataset | DBNDD1.group.cls#h\_versus\_l |
| Phenotype | group.cls#h\_versus\_l |
| Upregulated in class | l |
| GeneSet | KEGG\_ADHERENS\_JUNCTION |
| Enrichment Score (ES) | -0.32956386 |
| Normalized Enrichment Score (NES) | -1.2886089 |
| Nominal p-value | 0.22908367 |
| FDR q-value | 0.73362106 |
| FWER p-Value | 0.965 |
Table: GSEA Results Summary

  

Fig 1: Enrichment plot: KEGG\_ADHERENS\_JUNCTION      
 Profile of the Running ES Score & Positions of GeneSet Members on the Rank Ordered List

  

| SYMBOL | TITLE | RANK IN GENE LIST | RANK METRIC SCORE | RUNNING ES | CORE ENRICHMENT || 1 | RAC3 | na | 1824 | 0.312 | 0.0059 | No |
| 2 | TGFBR2 | na | 4515 | 0.197 | -0.0348 | No |
| 3 | PTPRM | na | 6543 | 0.136 | -0.0677 | No |
| 4 | SMAD2 | na | 7383 | 0.116 | -0.0701 | No |
| 5 | BAIAP2 | na | 7957 | 0.105 | -0.0670 | No |
| 6 | IGF1R | na | 8164 | 0.100 | -0.0541 | No |
| 7 | CREBBP | na | 9242 | 0.082 | -0.0698 | No |
| 8 | MAPK3 | na | 9865 | 0.073 | -0.0740 | No |
| 9 | TJP1 | na | 11569 | 0.052 | -0.1135 | No |
| 10 | CTNNA2 | na | 11866 | 0.048 | -0.1129 | No |
| 11 | SMAD4 | na | 11961 | 0.047 | -0.1067 | No |
| 12 | ACTN4 | na | 12890 | 0.039 | -0.1262 | No |
| 13 | PTPRJ | na | 13038 | 0.038 | -0.1233 | No |
| 14 | ERBB2 | na | 13132 | 0.037 | -0.1190 | No |
| 15 | SORBS1 | na | 13647 | 0.033 | -0.1277 | No |
| 16 | ACP1 | na | 14176 | 0.029 | -0.1375 | No |
| 17 | SNAI1 | na | 14205 | 0.028 | -0.1330 | No |
| 18 | INSR | na | 14612 | 0.026 | -0.1399 | No |
| 19 | ACTB | na | 14636 | 0.025 | -0.1358 | No |
| 20 | PTPN1 | na | 14723 | 0.025 | -0.1336 | No |
| 21 | RAC1 | na | 14759 | 0.025 | -0.1300 | No |
| 22 | NLK | na | 15329 | 0.020 | -0.1426 | No |
| 23 | WASF2 | na | 16020 | 0.016 | -0.1595 | No |
| 24 | PARD3 | na | 16208 | 0.014 | -0.1622 | No |
| 25 | CSNK2A1 | na | 16316 | 0.014 | -0.1627 | No |
| 26 | CTNNB1 | na | 16378 | 0.013 | -0.1620 | No |
| 27 | FARP2 | na | 16676 | 0.011 | -0.1684 | No |
| 28 | CTNND1 | na | 17239 | 0.008 | -0.1831 | No |
| 29 | IQGAP1 | na | 17318 | 0.007 | -0.1840 | No |
| 30 | ACTN2 | na | 17576 | 0.006 | -0.1903 | No |
| 31 | CSNK2A2 | na | 17761 | 0.005 | -0.1948 | No |
| 32 | WAS | na | 18085 | 0.002 | -0.2037 | No |
| 33 | ACTG1 | na | 18138 | 0.002 | -0.2049 | No |
| 34 | CTNNA1 | na | 18276 | 0.001 | -0.2087 | No |
| 35 | CDH1 | na | 18305 | 0.001 | -0.2094 | No |
| 36 | WASF1 | na | 19038 | -0.005 | -0.2296 | No |
| 37 | RHOA | na | 19096 | -0.005 | -0.2303 | No |
| 38 | CSNK2B | na | 19607 | -0.010 | -0.2432 | No |
| 39 | SMAD3 | na | 19999 | -0.013 | -0.2520 | No |
| 40 | TCF7L2 | na | 20073 | -0.014 | -0.2516 | No |
| 41 | WASL | na | 20105 | -0.014 | -0.2499 | No |
| 42 | RAC2 | na | 20865 | -0.021 | -0.2678 | No |
| 43 | LMO7 | na | 21236 | -0.024 | -0.2740 | No |
| 44 | VCL | na | 21288 | -0.025 | -0.2708 | No |
| 45 | YES1 | na | 21708 | -0.029 | -0.2774 | No |
| 46 | EGFR | na | 22041 | -0.033 | -0.2809 | No |
| 47 | CDC42 | na | 22626 | -0.039 | -0.2905 | No |
| 48 | MAPK1 | na | 22705 | -0.040 | -0.2853 | No |
| 49 | MAP3K7 | na | 23011 | -0.043 | -0.2860 | No |
| 50 | TCF7 | na | 23571 | -0.051 | -0.2926 | No |
| 51 | PTPRF | na | 23791 | -0.054 | -0.2889 | No |
| 52 | WASF3 | na | 24176 | -0.059 | -0.2890 | No |
| 53 | EP300 | na | 24292 | -0.060 | -0.2810 | No |
| 54 | PTPN6 | na | 24495 | -0.063 | -0.2749 | No |
| 55 | TGFBR1 | na | 26390 | -0.095 | -0.3117 | Yes |
| 56 | SSX2IP | na | 26509 | -0.098 | -0.2967 | Yes |
| 57 | TCF7L1 | na | 26813 | -0.104 | -0.2859 | Yes |
| 58 | CTNNA3 | na | 27076 | -0.110 | -0.2729 | Yes |
| 59 | FER | na | 27545 | -0.120 | -0.2640 | Yes |
| 60 | SRC | na | 28777 | -0.151 | -0.2712 | Yes |
| 61 | MET | na | 29439 | -0.170 | -0.2583 | Yes |
| 62 | ACTN1 | na | 30298 | -0.197 | -0.2460 | Yes |
| 63 | FYN | na | 31175 | -0.227 | -0.2286 | Yes |
| 64 | SNAI2 | na | 31822 | -0.255 | -0.1993 | Yes |
| 65 | LEF1 | na | 32857 | -0.317 | -0.1696 | Yes |
| 66 | PTPRB | na | 33448 | -0.366 | -0.1179 | Yes |
| 67 | FGFR1 | na | 33845 | -0.409 | -0.0525 | Yes |
| 68 | ACTN3 | na | 33884 | -0.415 | 0.0244 | Yes |
Table: GSEA details [plain text format]

  

Fig 2: KEGG\_ADHERENS\_JUNCTION      
 Blue-Pink O' Gram in the Space of the Analyzed GeneSet

  

Fig 3: KEGG\_ADHERENS\_JUNCTION: Random ES distribution      
 Gene set null distribution of ES for **KEGG\_ADHERENS\_JUNCTION**
